# Comparison of a Point-of-Care Analyzer With a Chemiluminescent Immunoassay for Serum Progesterone Measurement in Breeding Management of the Bitch

**DOI:** 10.3389/fvets.2021.660923

**Published:** 2021-05-13

**Authors:** Julia Zuercher, Katie M. Boes, Orsolya Balogh, Alyssa B. Helms, Julie T. Cecere

**Affiliations:** ^1^Virginia-Maryland College of Veterinary Medicine, Blacksburg, VA, United States; ^2^Department of Biomedical Sciences and Pathobiology, Virginia-Maryland College of Veterinary Medicine, Blacksburg, VA, United States; ^3^Department of Small Animal Clinical Sciences, Virginia-Maryland College of Veterinary Medicine, Blacksburg, VA, United States

**Keywords:** P4, in-house analyzer, IDEXX, Catalyst, analytical performance, validation, dog, Immulite

## Abstract

Accurate serum progesterone measurements for timing bitches during breeding management is critical for reproductive practice, especially as artificial insemination has become routine to facilitate breeding of animals that are geographically or temporally separated. To measure serum progesterone, chemiluminescent immunoassay (CLIA) has replaced radioimmunoassay as the current standard in the bitch due to its high correlation and increased practicality. In January 2019, a colorimetric point-of-care (POC) immunoassay for quantitative in-clinic canine serum progesterone measurements in <30 min was released. This study provides an independent comparison of the POC (Catalyst One, IDEXX) to the current industry standard, CLIA (Immulite-2000, Siemens). To assess inter-assay imprecision of POC and agreement of the POC and CLIA results, 100 canine serum samples were analyzed on three analyzers (POC-1, POC-2, and CLIA), of which, 74 (POC-1) and 75 (POC-2) results were within POCs' reportable range of 0.2–20 ng/mL and included in the study. To assess intra-assay imprecision, pooled canine serum samples at low (L1), intermediate (L2), and high (L3) progesterone concentrations were analyzed ten times each on POC-1 and CLIA. Relative to CLIA, POC values showed good correlation (POC-1, *r*^2^ = 0.9366; POC-2, *r*^2^ = 0.9438, *P* < 0.0001) and significant positive proportional bias at values >2 ng/mL. The POC inter-assay coefficients of variation (CVs) were 13.2% (0.2–2.9 ng/mL, 0.6–9.2 nmol/L, L1), 10.0% (3.0–9.9 ng/mL, 9.5–31.5 nmol/L, L2), 7.1% (10.0–20.0 ng/mL, 31.8–63.6 nmol/L, L3), and 11.2% (all samples). The intra-assay CVs for POC (L1, 15.3%; L2, 7.0%; L3, 4.7%) were higher than those for CLIA (L1, 5.89%; L2, 4.89%; L3, 3.44%). Based on the more rapid increase in serial serum progesterone concentrations in ovulating bitches and the greater imprecision of the POC, the clinical interpretations of serum progesterone measurements as they relate to canine breeding management should be made with caution.

## Introduction

Bitches are monoestrous with four distinct phases of their reproductive cycle, i.e., proestrus, estrus, diestrus, and anestrus. Follicle-stimulating hormone (FSH) is relatively high during the whole length of anestrus and decreases toward its end, while increasing frequency and amplitude of luteinizing hormone (LH) triggers the onset of proestrus (1–3). In response to FSH, developing follicles produce estrogen, which peaks during the proestrus phase. The bitch is unique in that luteinization of antral follicles results in measurable peripheral progesterone (P4) concentrations starting during proestrus, and increasing from basal values to 1.6–2.6 ng/mL at the time of the preovulatory LH peak. Following the preovulatory LH surge, follicular P4 production increases further and ovulation occurs 48–72 h later at approximately 5 ng/mL (between 4 and 10 ng/mL) serum P4 levels (1–7). The bitch ovulates immature oocytes arrested in prophase of the first meiotic division (germinal vesicle stage, GV) (1, 2, 5). After ovulation, peripheral P4 levels increase rapidly, which is thought to stimulate GV arrested oocytes to resume meiotic division and become secondary oocytes (arrested in Metaphase II) 48–72 h later (2, 5). Only secondary oocytes can be fertilized, which, together with their limited viability, determines a short fertilization period and thus a tight window of peak fertility (5, 6, 8). Due to the unique nature of their reproductive physiology, timing of breeding in the bitch is challenging.

Accurate serum P4 measurements to determine the day of the preovulatory LH peak and timing ovulation during canine breeding management is critical to maximizing pregnancy rates and litter size. Improper timing of breeding is the number one cause of infertility in the bitch (8). Because breeding animals are often geographically or temporally separated, artificial insemination with fresh, shipped, chilled or frozen semen has become routine in reproductive practice. The varied window of peak fertility, combined with the reduced longevity of semen that has been frozen or chilled for shipping purposes, poses a problem to most veterinarians timing bitches for breeding (9, 10).

Gestation length in the bitch is well-documented to be 63 ± 2 days from ovulation, although breed differences also seem to play a role (11–16). Consequently, determination of ovulation date through serum P4 measurements during breeding allows veterinarians to calculate the expected parturition date and prepare for the whelping, manage a small litter or high-risk pregnancies. After ovulation, corpora lutea produce high quantities of P4. In the dog, the corpus luteum is the sole source of P4 during diestrus. Peak serum P4 concentrations between 15 and 80 ng/mL are reached during the first half of the luteal phase between 20 and 35 days post-ovulation (11), after which serum P4 steadily declines during the second half of diestrus (15, 16). Prolactin concentrations increase during the second half of the luteal phase and have a major luteotrophic effect (2, 4, 16, 17). In pregnant bitches, placental production of relaxin as early as day 24 post-ovulation enters peripheral circulation and is suspected to stimulate prolactin secretion, perhaps indirectly having luteotrophic effects for maintenance of pregnancy (17–20). At term pregnancy, as the fetal pituitary-adrenal axis matures and fetal cortisol is released, placental prostaglandin F2α production initiates luteolysis (2, 14–16, 21). Prostaglandin F2α metabolite concentrations peak around 36 h prior to parturition, and as luteolysis ensues, serum P4 concentrations drop below 2 ng/mL ~24–36 h before parturition (2, 21). Due to P4's thermogenic properties, its abrupt decrease leads to a transient temperature drop of 1 degree Celsius approximately 24 h prior to whelping, although this is not observable in all bitches (21).

In addition to its use for breeding management, serum P4 measurement is a critical tool throughout gestation and around parturition for monitoring high-risk pregnancies and determining the timing of intervention if needed. Hypoluteoidism or luteal insufficiency results in a premature drop in serum P4, leading to embryonic resorption or abortion of fetuses (22–24). Progesterone supplementation may become necessary to maintain the pregnancy and must be initiated when serum P4 falls below 5 ng/mL prior to day 58–60 from ovulation (22–24). The pregnancy is at risk of failure if serum P4 falls below 2 ng/mL, which is the threshold for pregnancy maintenance (11, 13, 15). For this reason, accurate serum P4 measurement is essential to monitor high-risk pregnancies, because even small errors could preclude the bitch from treatment and thus risk pregnancy loss. Additionally, “reverse P4 timing” at the end of gestation, i.e., daily or every other day serum P4 measurements starting 5–7 days before the expected day of parturition, can be used together with ultrasonographic evaluation of fetal maturation (13, 25, 26) to determine when a Cesarean section (C-section) can be safely performed. Puppies delivered prematurely may not be sufficiently developed for survival, and puppies delivered too late could outlive their placental nutrient supply and die *in utero* (12, 13). Accurate timing of C-section is critical when the date of ovulation is unknown, especially in breeds that have difficulty whelping naturally, such as brachycephalic dogs, or in bitches carrying small litters (singleton or two-pup pregnancies) where placental prostaglandin F2α release may be insufficient to cause timely prepartal drop of P4.

Serum P4 measurements in general and specialty practice have become routine for managing breeding, high-risk pregnancies, elective and planned C-sections, and dystocias. Additionally, serum P4 measurements can be used to determine spay status in the bitch when paired with luteinizing hormone (LH) or anti-Müllerian hormone (AMH) assays, as well as guide therapies for reproductive disorders, such as pyometra and cycle abnormalities (27–29). Although P4 is a highly conserved molecule across species, laboratory methods for measuring serum P4 concentrations in the dog vary in accuracy and precision. Radioimmunoassay (RIA) is the historical industry-standard for canine serum P4 measurement but has limited availability. Due to its inherent radiation hazard, equipment cost, decreased availability of reagents, and result reporting of 24 h or longer, very few reference laboratories currently offer serum P4 measurements via RIA in dogs (30, 31). Consequently, chemiluminescent immunoassay (CLIA), which uses fluorescence and anti-P4 antibodies, has become the contemporary industry standard offered by reference laboratories due to its lower cost and faster turnaround time compared to RIA (30–33). A comparative study of RIA and CLIA measurements of canine serum P4 concentration determined the two tests to be highly correlated (*r* = 0.9709; *P* < 0.0001) (9). Furthermore, studies on serum P4 concentration in the bitch using CLIA have shown the molecule to be highly stable. No significant concentration differences were seen between separated vs. unseparated plasma, separated plasma stored at room temperature for 14 days, and samples subjected to up to 10 freeze-thaw cycles (34).

To meet the demand for affordable, same-day semi-quantitative P4 results, many point-of-care analyzers (POCs) have been released, including the MiniVidas (Biomerieux, France) and the AIA-360 (Tosoh Corp., Japan) automated analyzers. Intended for human use, MiniVidas uses enzyme-linked fluorescence assay (ELFA) technology, and calculates semi-quantitative P4 measurements within 45 min. This system showed a significantly high correlation (*r* = 0.989) to the RIA standard in bitches over the range of clinically significant P4 values, and was reliably parallel to clinical findings (35). The AIA-360 analyzer uses fluorescent enzyme immunoassay (FEIA) to quantify serum P4 concentration. To establish validity, the AIA-360 P4 results were compared to liquid chromatography and mass spectrometry (LC-MS), which “has been proposed as a gold standard” for steroid hormone measurements but has yet to be validated for quantitative canine P4 assays (36–40). While there was strong a significant correlation (*r* = 0.979; *P* < 0.001) between P4 measurements obtained on the AIA-360 FEIA with LC-MS, FEIA values were significantly higher than the LC-MS comparison (36). A high correlation (*r*^2^ = 0.978) has been illustrated between the AIA-360 analyzer and the CLIA standard (41). Unfortunately, the intervariance of these in-clinic analyzers has posed a large problem for breeding and artificial insemination timing (33).

In January 2019, a colorimetric immunoassay for two well-established chemistry POCs was released, providing serum P4 results in 12 min. As many small animal general and specialty veterinary practitioners need same-day or even same-hour quantitative serum P4 measurements, this POC has great potential to provide valuable information quickly. While manufacturer-issued literature states “very good correlation to the reference method,” the POC's P4 assay has only been compared to liquid chromatography and mass spectrometry (LC-MS) and not to the more widely used CLIA, which is currently regarded as the industry standard for canine serum P4 measurements (37, 38). To date, no independent validation has been performed on this POC assay. Although preliminary data has been collected comparing CLIA and LC-MS in endogenous steroid hormones in the bitch, there is a need for more extensive comparative data between serum P4 measurements by CLIA and LC-MS (38, 42). Even a small bias in serum P4 values can lead to missed cycles or breedings, decreased pregnancy rates and litter sizes, inaccurate whelping date calculations, or a different course of treatment in certain reproductive diseases or conditions.

This study aimed to compare canine serum P4 measurements between POC and CLIA to assess the agreement of POC as an in-clinic diagnostic tool, and to determine intra- and inter-assay precision for POC. Despite its speed and accessibility, we hypothesized the differences between the two methods would affect the agreement of canine serum P4 results within the clinical range of interest.

## Materials and Methods

### Analyzers and Assays

The CLIA, which uses fluorescence and anti-P4 antibodies, was maintained per the manufacturer's recommendations as the reference method (Immulite-2000, Siemens Medical Solutions USA, Inc., Malvern, Pennsylvania, USA).

Two in-clinic competitive, enzyme-based, colorimetric, P4 immunoassay POCs were maintained per the manufacturer's recommendations (Catalyst One, IDEXX Laboratories, Inc, Westbrook, Maine, USA). For this study, the two analyzers will be referred to as POC-1 and POC-2.

### Samples

#### Sample Sources and Demographics

One hundred serum samples were collected from 35 bitches that were presented to Virginia-Maryland College of Veterinary Medicine's Veterinary Teaching Hospital for reproductive services between November 2019 and July 2020. Eleven samples were diestral with ten mid-diestral samples collected at pregnancy confirmation ultrasound and one late-diestral sample collected 51 days after ovulation. The remaining 89 were estrual, which were further categorized by their day of estrus with day one defined as the first day of serosanguineous vulvar discharge. Twenty-seven samples were collected during the first week of estrus (days 1–7), 48 were collected during the second week (days 8–14), and 14 samples were collected during the third week (days 15–21). Only samples with sufficient serum volume to be analyzed on the three analyzers were included in the study. The 35 bitches represented 19 breeds: seven German Shepherd Dogs, four Golden Retrievers, three Labrador Retrievers, two Cavalier King Charles Spaniels, two English Bulldogs, two Irish Wolfhounds, two Leonbergers, two Newfoundlands, and one bitch from each of the following breeds: Afghan Hound, Bullmastiff, Coonhound, Doberman Pinscher, English Shepherd, French Bulldog, Great Dane, Lowchen, Polish Lowland Sheepdog, Stabyhoun, and Weimaraner.

#### Method Comparison and Inter-assay Precision

Samples were analyzed on CLIA, and then immediately frozen at −20°C (−4°F) until batch and randomized analysis on POC-1 and POC-2 could be performed in August 2020, at which time the serum was thawed at room temperature at 20–22°C (68–72°F) for approximately 15 min before recording for the presence of lipemia, icterus, or hemolysis and immediate analysis. After analysis, the samples were refrozen at −20°C (−4°F) and stored for the intra-assay precision study. Samples were excluded from statistical analysis if the P4 concentration was below or above the POC's reportable range [ <0.2 or >20.0 ng/mL (<0.6 or >63.6 nmol/L)]. Of the 100 serum samples, the CLIA progesterone readings were as follows: 27 samples with <1 ng/ml, 24 samples between 1 and 4 ng/ml, 22 samples between 4 and 10 ng/ml, and 27 samples with >10 ng/ml progesterone concentration.

#### Intra-assay Precision

Using CLIA results, serum samples were identified to be pooled at three P4 levels: L1, 1.00–2.00 ng/mL (3.18–6.36 nmol/L); L2, 5.8–7.8 ng/mL (18.44–24.80 nmol/L), and L3, 8.0–13.0 ng/mL (25.44–41.34 nmol/L). Serum samples were thawed at room temperature at 20–22°C (68–72°F) for 15 min, pooled by level, mixed on a rocker for 5 min, separated into 20 aliquots, and immediately analyzed or stored at −20°C (−4°F) until analysis. Each pooled sample was analyzed ten consecutive times on CLIA and POC-1.

### Statistical Analysis

Statistical analysis was performed using JMP Pro 15.0.0 (SAS Institute, Inc., Cary, North Carolina, USA). Data set were assessed for normality using the Shapiro-Wilk W test with statistical significance set at *p* ≦ 0.05. Means, standard deviations (SD), 95% confidence interval (CI), and result ranges were reported for parametric data sets, whereas medians, interquartile ranges, and result ranges were reported for non-parametric data sets.

#### Method Comparison

Pearson's correlation, Passing-Bablok regression, and Bland-Altman analysis were performed, which included using an Add-In for the Passing-Bablok and Bland-Altman analyses (Method Comparison.jmpaddin, https://community.jmp.com/t5/JMP-Add-Ins/Method-Comparison/ta-p/21520). Serial serum P4 concentrations from bitches monitored for ovulation were plotted over time.

#### Precision

The inter-assay coefficient of variation (CV) was used to assess inter-assay imprecision. First, individual sample standard deviations (SDs) and CVs were determined: individual sample CV (%) = SD of duplicate POC-1 and POC-2 sample results/mean of duplicate POC-1 and POC-2 sample results x 100. The intra-assay SDs and CVs were then calculated by averaging the individual sample SDs and CVs, respectively, at low (L1: 0.2–2.9 ng/mL, 0.6–9.2 nmol/L), intermediate (L2: 3.0–9.9 ng/mL, 9.5–31.5 nmol/L), high (L3: 10.0–20.0 ng/mL, 31.8–63.6 nmol/L), and all inter-assay mean concentrations.

Intra-assay imprecision was assessed at L1, L2, and L3 by calculating the SD and using the CV formula: CV (%) = SD of ten replicates/mean of ten replicates ×100.

## Results

### Samples

Of the 100 samples that met the inclusion criteria of the study, 25 samples were excluded from POC-1's data set due to results being below (*n* = 3) or above (*n* = 22) the analyzer's reportable range, whereas 26 samples were excluded from POC-2's data set for results being below (*n* = 4) or above (*n* = 22) the reportable range. Samples that were excluded had CLIA P4 concentrations of 0.20–0.24 ng/mL (0.64–0.76 nmol/L) or 13.00–30.50 ng/mL (41.34–96.99 nmol/L). Descriptive statistics for the three data sets are provided in Table 1. Seventy-seven samples were grossly evaluated for the presence or absence of lipemia, icterus, or hemolysis, which identified 5 lipemic samples, 4 icteric samples, 55 hemolyzed samples, and 23 samples with a normal serum appearance.

**Table 1:** Descriptive statistics for progesterone concentrations obtained from 100 canine serum samples analyzed using a reference chemiluminescent immunoassay (CLIA) and two point-of-care analyzers (POC-1 and POC-2).

**Method**	**N**	**Mean ± SD**	**95% CI of the Mean**	**Range**
CLIA	100	6.77 ± 7.19 ng/mL	5.34–8.20 ng/mL	0.20–30.50 ng/mL
		21.54 ± 22.87 nmol/L	17.00–26.07 nmol/L	0.64–96.99 nmol/L
POC-1	75	4.5 ± 4.8 ng/mL	3.4–5.7 ng/mL	0.2–19.8 ng/mL
		14.4 ± 15.6 nmol/L	10.8–18.0 nmol/L	0.6–59.8 nmol/L
POC-2	74	5.2 ± 5.6 ng/mL	3.9–6.5 ng/mL	0.2–18.8 ng/mL
		16.7 ± 17.9 nmol/L	12.5–20.8 nmol/L	0.6–63.0 nmol/L

*CI, confidence interval; N, sample number; SD, standard deviation*.

### Method Comparison

**Figure 1:**
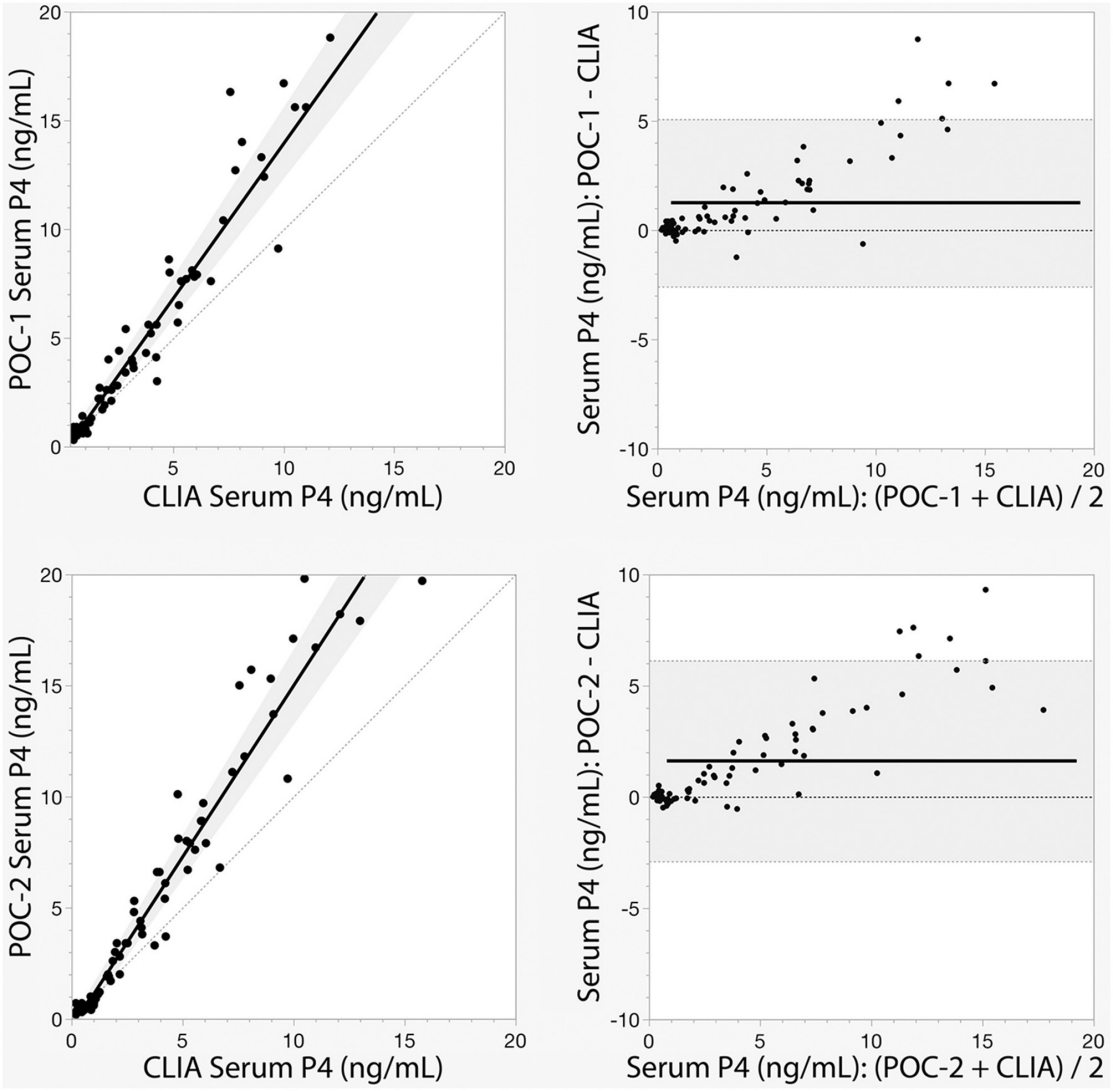
Passing-Bablok regression (left) and Bland-Altman (right) graphs of canine serum progesterone (P4) concentrations measured by two point-of-care analyzers (POC-1 and POC-2) and a reference chemiluminescent immunoassay (CLIA). Passing-Bablok regression graphs show the black lines of best fit flanked by the shaded 95% confidence curves, and plotted with the gray line of identity (y = x). Bland-Altman bias graphs depict the black mean bias line with the shaded 95% limits of agreement lines; the gray line of no bias (*y* = 0) is also shown.

**Figure 2:**
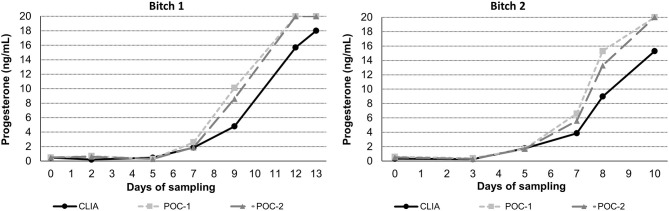
Representative serum progesterone curves measured by the reference chemiluminescent immunoassay (CLIA) and two point-of-care analyzers (POC-1 and POC-2) from two individual bitches during breeding management. Day 0 is the day blood sampling started, which corresponded to day 5–7 from the first day of proestrus. Progesterone concentrations above the upper limit of detection of the POC (20 ng/mL) are depicted as 20 ng/mL.

Pearson's correlation coefficients (R) and coefficients of determination (R^2^) were 0.9678 and 0.9366 for POC-1 by CLIA (*P* < 0.0001), 0.9551 and 0.9438 for POC-2 by CLIA (*P* < 0.0001), and 0.9879 and 0.9759 for POC-1 by POC-2 (*P* < 0.0001), respectively. Datasets between both POCs and the CLIA showed good correlation, significant positive proportional biases, and no significant constant biases (Figure 1 and Table 2). Analysis of the 23 samples with no lipemia, icterus, or hemolysis produced similar statistical results to those of the complete datasets (results not shown). Serial serum P4 concentrations from bitches showed POC's serum P4 concentrations increasing more rapidly than those from CLIA (Figure 2).

**Table 2:** Passing-Bablok regression and Bland-Altman analytics of canine serum progesterone concentrations measured by two point-of-care analyzers (POC-1 and POC-2) and a reference chemiluminescent immunoassay (CLIA).

**Comparison**	**N**	***t***	**Slope (95% CI)**	**Intercept (95% CI)**	**Mean bias (95% LOA)**
POC-1 by CLIA	75	0.8594	1.42 (1.29–1.56)	−0.21 ng/mL (−0.57–0.03 ng/mL)	1.24 ng/mL (−2.59–5.07 ng/mL)
				−0.67 nmol/L (−1.81–0.10 nmol/L)	3.94 nmol/L (8.24–16.12 nmol/L)
POC-2 by CLIA	74	0.8790	1.54 (1.39–1.67)	−0.37 ng/mL (−0.70–0.08 ng/mL)	1.61 ng/mL (−2.90–6.13 ng/mL)
				−0.89 nmol/L (−2.29–0.51 nmol/L)	5.12 nmol/L (9.22–19.49 nmol/L)

*CI, confidence interval; LOA, limits of agreement; N, sample number; t, correlation coefficient*.

### Precision

Point-of-care analyzer's inter-assay CVs were 13.2% (L1), 10.0% (L2), 7.1% (L3), and 11.2% (all samples) (Table 3). Intra-assay CVs were 5.89% (L1), 4.89% (L2), and 3.44% (L3) for CLIA, and 15.3% (L1), 7.0% (L2), and 4.7% (L3) for POC (Table 4).

**Table 3:** Inter-assay coefficients of variation (CVs) at low (L1), intermediate (L2), high (L3), and all concentrations of canine serum progesterone for a point-of-care analyzer.

**Level**	**N**	**Mean**	**Range**	**SD**	**CV (%)**
L1	36	1.0 ng/mL	0.3–2.8 ng/mL	0.1 ng/mL	13.2
		3.18 nmol/L	1.0–8.9 nmol/L	0.3 nmol/L	
L2	25	6.0 ng/mL	3.1–9.4 ng/mL	0.5 ng/mL	10.0
		19.1 nmol/L	9.9–29.9 nmol/L	1.6 nmol/L	
L3	11	14.6 ng/mL	10.0–18.5 ng/mL	1.0 ng/mL	7.1
		46.4 nmol/L	31.8–58.8 nmol/L	3.2 nmol/L	
All	72	4.8 ng/mL	0.3–18.5 ng/mL	0.4 ng/mL	11.2
		15.3 nmol/L	1.0–58.8 nmol/L	1.3 nmol/L	

*N, sample number; SD, standard deviation*.

**Table 4:** Intra-assay coefficients of variation (CVs) at low (L1), intermediate (L2), and high (L3) concentrations of canine serum progesterone for a point-of-care analyzer (POC) and reference chemiluminescent assay (CLIA).

**Level**	**N**	**Mean**	**SD**	**CV (%)**
L1 (CLIA)	10	2.12 ng/mL	0.12 ng/mL	5.89
		6.74 nmol/L	0.40 nmol/L	
L1 (POC)	10	1.4 ng/mL	0.2 ng/mL	15.3
		4.3 nmol/L	0.7 nmol/L	
L2 (CLIA)	10	7.58 ng/mL	0.37 ng/mL	4.89
		24.10 nmol/L	1.18 nmol/L	
L2 (POC)	10	12.3 ng/mL	0.9 ng/mL	7.0
		39.2 nmol/L	2.8 nmol/L	
L3 (CLIA)	10	10.30 ng/mL	0.35	3.44
		32.74 nmol/L	1.13 nmol/L	
L3 (POC)	10	14.9 ng/mL	0.7 ng/mL	4.7
		47.3 nmol/L	2.2 nmol/L	

*N, sample number; SD, standard deviation*.

## Discussion

The release of the colorimetric POC P4 immunoassay had great promise as an in-clinic diagnostic tool for reproductive management and diagnostics in the bitch. Manufacture-issued literature states “very good correlation to the reference method of LC-MS” (37, 38) for measuring serum P4. The manufacturer's literature also states, “LC-MS has been proposed as the gold standard” (37, 38) due to the small sample volume requirement, faster testing time, and higher specificity; however, the current lack of standardization and commercialization of steroid hormone LC-MS assays poses a major obstacle to its clinical utility (37, 39, 40, 43). The reference cited by the manufacturer for this proposed industry standard reviews LC-MS as a measure of human serum estrogen concentrations; serum P4 was not assessed (40). Although a few studies have preliminarily compared serum P4 values by LC-MS to RIA or CLIA in the bitch, validated LC-MS canine serum P4 reference intervals or clinical decision limits have not been assessed in many contexts (38, 39, 41, 42). Subsequently, the clinical utility of LC-MS to measure canine serum P4 is limited (39, 40, 42, 43). Historically, RIA has been regarded as the industry standard for canine serum P4 measurements, and correlates well to the more widely used CLIA (30–33), the current industry standard used by most veterinary reference laboratories (30–33, 37, 38). Therefore, it is more clinically appropriate to compare the performance of POC to CLIA, as performed in this study.

Although the correlations between the two POCs and CLIA were good, the POC and CLIA produced divergent results with differences between values increasing as serum P4 concentration increased. This lack of agreement is demonstrated in the regression and bias analyses (Table 2 and Figure 1), as well as representative serum P4 curves (Figure 2). The difference became more evident at and above serum P4 concentrations of 2 ng/mL (Figures 1, 2), and values >10 ng/mL begin to fall outside of the 95% LOAs (Figure 1). With the upper end of the 95% LOAs at a difference of 5 ng/mL between POC-1 and CLIA, and 6 ng/mL between POC-2 and CLIA (Figure 1), different clinical interpretations could result from these analytical discrepancies. As shown in Figure 2, the representative curves from each of two bitches plotted CLIA and POCs' serum P4 concentrations throughout breeding management. The curves by POC-1 and POC-2 consistently rose faster than those of CLIA. This earlier rise in serum P4 could lead to anticipated ovulation 1–2 days sooner than the actual ovulation, thus predicting insemination dates outside of the fertile window. For insemination with frozen semen, for which the window for fertilization is especially narrow (8), it is likely breeding management will be mistimed and unsuccessful if curves derived from POC are used for interpretation.

Analytic imprecision was assessed by the intra-assay and inter-assay CVs; the greater the CV, the greater the dispersion of results around the mean, and the lower the repeatability of a result. The desirable specification for serum P4 imprecision has not been established in bitches, but the desirable analytical CV for human assays is ≤9.8% and tends to be ≤10.0% for most steroid assays (44). Point-of-care analyzer's intra-assay CVs were ≤10.0% at two of the three concentrations assessed and were greater than CLIA's intra-assay CVs at all three levels, respectively. This is in contrast to POC's manufacturer's claim that “the new method of…progesterone [measurement] had a total CV of <10% at both concentration levels” (37, 38). This discrepancy may be attributed to POC's inter-assay imprecision (inter-assay CV of 11.2%), and therefore differences in repeatability of results between two different POCs, or because different samples were analyzed in the manufacturer's study and this study. The manufacturer opted to perform a repeat analysis of quality control material (derived from an unspecified species or manufacturing method), whereas repeat analysis performed in this study analyzed pooled canine serum. Of these two samples, pooled canine serum is more representative of clinical samples from bitches, and would therefore produce more accurate CV results due to quality control material's potential matrix effects. In contrast, CLIA's intra-assay CVs were <6.0 ng/mL at all three serum P4 concentrations, well below the desirable analytical CV for human assays.

The faster rise in POC's serum P4 curve may lead to more difficult detection of smaller differences in analyte concentrations. When combined with the greater imprecision, using the POC to detect clinical trends can be problematic. When managing high-risk pregnancies, for example, P4 supplementation must be started if the bitch falls below 5 ng/mL prior to day 58 to 60 from ovulation (22–24). Given the results illustrated in Figure 1, the POC is unlikely to detect this critical threshold and could lead to untreated pre-term labor and abortion. With regard to breeding management, precise serum P4 measurements with known reference intervals are essential for determining ovulation as the transition from primary (immature) to secondary (mature) follicles occurs 48–72 h after ovulation has occurred (2, 5). Because only secondary oocytes can be fertilized (5, 6, 8), the short window of fertility could be missed with the P4 measurements do not properly detect ovulation.

Based on the data collected in this study, (1) POC has good agreement with CLIA when serum P4 is <2 ng/mL, which does not include the entire clinical range of interest (0.2–20.0 ng/mL), (2) serial serum P4 concentrations increase more rapidly when using POC compared to CLIA, and (3) POC is more imprecise than CLIA. Based on these findings, POC's serum P4 assay has limited utility for canine breeding management until further studies are performed. However, the assay may be useful when only serum P4 concentrations of 0.2–2.0 ng/mL are needed, such as dystocia management or clinical decision-making on diestral disease management (e.g., pyometra, determining treatment endpoints), or when qualitative serum P4 measurements are sufficient >2.0 ng/mL.

## Concluding Remarks

This paper presents an independent validation of chromogenic point-of-care (POC) progesterone (P4) immunoassay in comparison to chemiluminescent immunoassay (CLIA). Chemiluminescent immunoassay highly correlates with and is more practical than radioimmunoassay (RIA), the historical industry standard for serum P4 measurements in the bitch. Based on the results, the significant positive proportional bias of POC compared to CLIA became evident at and above 2 ng/mL, with P4 concentrations starting to fall out of the 95% limits of agreement above 10 ng/mL. Furthermore, POC has an acceptable imprecision at higher P4 concentrations, but imprecision falls outside the acceptable range at low serum P4 concentrations. Because discernable and precise serum P4 concentrations in the clinical range (0.2–20 ng/mL) are essential for ovulation timing and determination of optimal mating/breeding dates as well as for clinical decision making on treatments of various reproductive conditions, the findings limit the use of POC by reproductive veterinarians. Until further validation is performed on liquid chromatography and mass spectrometry as a means of measuring serum P4 in the bitch, we recommend that future POC analyzers should be compared to RIA or CLIA before commercial release.

## Data Availability Statement

The raw data supporting the conclusions of this article will be made available by the authors upon request, without undue reservation.

## Author Contributions

JC, OB, and KB: conceived and designed study. JC, OB, KB, and AH: collected, compiled, analyzed data, and edited manuscript. KB: statistical analyses. JZ: drafted and edited manuscript. All authors contributed to the article preparation and approved the submitted version.

## Conflict of Interest

The authors declare that the research was conducted in the absence of any commercial or financial relationships that could be construed as a potential conflict of interest.
